# Effect of Biologic Material Reinforcement on Surgical Anastomosis After Gastrectomy—A Pilot Study

**DOI:** 10.3389/fonc.2019.01184

**Published:** 2019-11-06

**Authors:** Won Jun Kim, Chang Min Lee, Liang An, Jong-Han Kim, Sungsoo Park

**Affiliations:** ^1^Korea University Medical Center, College of Medicine, Seoul, South Korea; ^2^Department of Surgery, Korea University Medical Center, Ansan Hospital, Ansan, South Korea; ^3^Department of Surgery, Korea University Medical Center, Guro Hospital, Seoul, South Korea; ^4^Department of Surgery, Korea University Medical Center, Anam Hospital, Seoul, South Korea

**Keywords:** anastomotic leak, surgical anastomosis, gastrectomy, postoperative complications, gastric cancer, acellular dermal matrix

## Abstract

**Background:** Acellular dermal matrix is a biologic material derived from the skin of human cadaveric donors. It has been used successfully in the past to reduce complications in breast surgery and hernia repair. This investigation was aimed at assessing the feasibility of using acellular dermal matrix to support the anastomosis after gastrectomy with the aim of reducing anastomotic site leakage complications.

**Methods:** Patients were randomly assigned to standard anastomotic reconstruction (control arm) or anastomotic reconstruction with acellular dermal matrix reinforcement (intervention arm). Surgical outcomes related to anastomotic complications were collected. Because actual anastomotic leaks found on imaging studies are infrequent and thus require a very high number of patient recruitment to detect statistically significant difference between the two groups, in this pilot investigation other clinical and laboratory measures that have been shown to correlate to or predict anastomotic leaks were also collected. Each surgical outcome was compared.

**Results:** A total of 94 patients (intervention arm: 50, control arm: 44), were included in the analysis. Two patients in the control arm (4.55%) and one patient in the intervention arm (2.00%) experienced anastomotic leakage (*p* = 0.598), a difference without statistical significance. However, average postoperative C-reactive protein (CRP) levels and NUn scores, both of which have been shown to reflect likelihood of progressing to anastomotic leakage, were significantly lower for the intervention arm. The control arm showed an average CRP level of 128.77 mg/dL (SD: 97.08) while the intervention arm showed 77.38 mg/dL (SD: 49.08, *p* = 0.049).

**Conclusions:** Leakage rate reduction with acellular dermal matrix reinforcement of anastomotic site was not detected in this investigation. However, postoperative inflammation levels and numerical predictors of anastomotic leakage development were significantly lower with acellular dermal matrix reinforcement of surgical anastomosis. This finding is worthy of further investigation, as reduction of inflammation with anastomotic site reinforcement is a novel finding, and more in-depth research may lead to discoveries on the physiologic role of the surgical anastomosis in post-gastrectomy patients. In addition, lower CRP and NUn scores for the intervention arm suggest potential for larger studies to detect reduction in clinical leak rates after acellular dermal matrix reinforcement.

## Introduction

Surgery of the gastrointestinal tract is most often concluded with anastomotic reconstruction of resection planes to restore gut continuity. Surgeons pay careful attention to these anastomotic sites as they are critical to procedure-related complications. The most dangerous and important anastomotic site complication is leakage, which is associated with increased morbidity and longer hospital stay as well as higher mortality (1). As the management of the anastomotic site during the reconstruction process is crucial in reducing these potentially fatal complications, the rates of such postoperative complications are often used as a surrogate marker for the quality of surgery (1–3). Although the surgical outcome for gastrointestinal surgeries has improved over time with experience and advancements in technique, the rate of postoperative complications remains high worldwide (4, 5). Various implements have been introduced in the surgical procedure, with the aim of reducing these complications: examples include the surgical stapler and bioabsorbable synthetic material scaffolds that support the anastomotic site (6–11). These new additions to the surgeon's arsenal have succeeded in reducing postoperative anastomotic complications to a certain degree (11, 12); but there is still much room for improvement, and various new approaches are being investigated by surgeons to further decrease anastomotic complication rates.

In contrast to the aforementioned bioabsorbable “synthetic” material, the acellular dermal matrix was developed as a “biologic” material scaffold for tissue reinforcement. The most prominent synthetic material reinforcement in use is the polyglycolic acid:trimethylene carbonate copolymer (Gore Seamguard®), which features an interconnected pore structure that allows the cells of the host to grow within it; it is then absorbed into host tissue around the staple line within six to seven months. It has been used to reduce postoperative complications in patients who have undergone gastrointestinal surgery. Biologic material like the acellular dermal matrix used in this investigation stands apart from the synthetic counterparts owing to its biologic origin. It is a connective tissue matrix of dermis harvested from the skin of human cadaveric donors, with the cellular components removed, based on the hypothesis that this may confer an advantage over synthetic material reinforcements as it can be revascularized with autologous tissue, resulting in reduced rates of infection and better maintenance of tissue strength, which have been shown to be true in animal studies (13, 14). Acellular dermal matrix is already being used in a number of applications, such as the repair of difficult hiatal hernias and the treatment of intestinal fistulization in patients with open peritoneal cavities (15, 16). The specific material used in this investigation (MegaDerm® - L&C Bio, SKN Techno Park, Sagimakgol-ro, Jungwon-gu, Seongnam-si, Gyeonggi-do, Korea) has also been used in plastic and reconstructive surgical applications (17–20). In this study, we aimed to assess the feasibility of using acellular dermal matrix reinforcement to reduce complications in patients who underwent gastrectomy, with a focus on anastomotic site leakage.

## Materials and Methods

### Study Design

This pilot investigation is a randomized, double-arm, open-label, superiority study conducted in a single institute. Patients who had undergone total or subtotal gastrectomy for gastric adenocarcinoma were enrolled in this study. Enrollment took place from July of 2015 to April of 2016. Patients who provided their informed consent were randomized to either the control arm or the intervention arm: randomization sequence was created using Excel 2010 (Microsoft, Redmond, WA, USA) with a 1:1 allocation using simple randomization without stratification. Patients who underwent total or subtotal gastrectomy followed by standard anastomotic reconstruction without acellular dermal matrix reinforcement comprised the control arm, while those who underwent gastrectomy with the acellular dermal matrix reinforcement comprised the intervention arm. Data collected from the patients were analyzed by an independent investigator who was unaware of the allocation of each patient. The study protocol was approved by the institutional review board of Korea University Medical Center, Anam Hospital (Institutional Review Board number: MD15006). All procedures were conducted in accordance with the ethical standards of the institution's Committee on Human Experimentation and the Helsinki Declaration of 1975.

### Patient Enrollment

The following inclusion and exclusion criteria were used for patient enrollment:

Inclusion criteria:

- Patients between the ages of 20 and 90 years;
- Patients who were diagnosed with primary gastric adenocarcinoma by endoscopic biopsy;
- Patients who were fit for total or subtotal gastrectomy;
  - ECOG (Eastern Cooperative Oncology Group) performance status 0 or 1
  - ASA (American Society of Anesthesiologists) score between 1 and 3
  - Patients who were not contraindicated for surgery based on the preoperative work-up
- Patients who provided informed consent by signing the IRB-approved consent form.

Exclusion criteria:

- Patients who developed complications of gastric cancer (i.e., obstruction, perforation);
- Patients who received neoadjuvant chemotherapy or radiation therapy for the target gastric cancer of the surgery;
- Patients who received surgical or medical treatment for any other cancers in the last 5 years;
- Vulnerable patients (patients who are unable to make their own decisions, pregnant patients, patients planning on getting pregnant);
- Patients who are currently or were enrolled in any time in the last 6 months in another clinical trial.

### Operative Procedures and Postoperative Management

**Figure 1:**
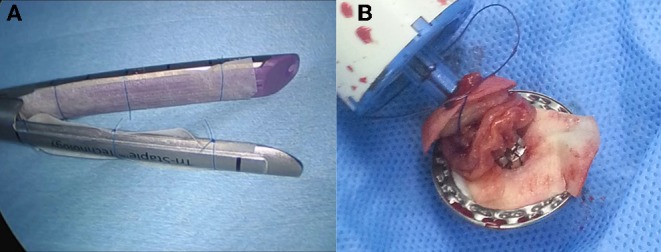
Acellular dermal matrix loaded for use **(A)** linear stapler **(B)** circular stapler.

Patients were treated according to the standard guidelines for treatment of gastric cancer in Korea (21), which outlines the principles and standards of surgery for gastric cancer. All cases were laparoscopic with no conversion to open laparotomy. In all patients, lymphadenectomy was facilitated by an ultrasonic energy device (SOUND REACH® Reach Surgical Inc., TEDA, Tianjin). Reconstruction procedures used were Billroth I, Billroth II, and Roux-en-Y esophagojejunostomy. Anastomotic reconstruction was conducted using the surgical stapler (ENDO REACH® Reach Surgical Inc., TEDA, Tianjin); for patients in the intervention arm, acellular dermal matrix (MegaDerm® - L&C Bio, SKN Techno Park, Sagimakgol-ro, Jungwon-gu, Seongnam-si, Gyeonggi-do, Korea) was installed onto the surgical stapler with a hygroscopic suture fiber before use, after being moisturized to increase adhesiveness (Figure 1). Postoperative care was provided according to the institution's protocols.

### Clavien-Dindo Classification Assessment

The main surgical outcome assessed in this investigation is the rate of anastomotic leakage. We primarily compared the severity of these postoperative anastomotic site complications in each group as measured by the modified Clavien-Dindo system, which is a widely adopted, objective classification system that grades the severity of surgical complications based on the level of intervention needed to resolve them (22). When the outlines proposed by the Clavien-Dindo classification were vague, the Japanese Clinical Oncology Group postoperative complications criteria (JCOG PC) criteria (23), which expands on the Clavien-Dindo system and more specifically delineates grades of each postoperative complication, were applied. As the potential for interpersonal variation remained, the classification process was conducted by two independent researchers who were unaware of each patient's treatment arm allocation. When discrepancies arose, the principal investigator (S.P.) was consulted to determine the final Clavien-Dindo classification for the patient.

### Anatomic Leakage Assessment

Computed tomography (CT), considered the best modality for the detection of gastrointestinal leakage (24), was the modality of choice when imaging was deemed clinically necessary. The clinical suspicion of leakage warranting imaging work-up was made by the patient's physician or the attending surgeon, with the basic consensus that episodes of fever peaking around 38.0°C constitute the most important clinical sign, as it has been designated a critical criterion for the suspicion of anastomotic leakage in previous studies (24–26). In addition, because post-gastrectomy leak rates are low and therefore difficult to statistically detect differences in leak rates between the two groups, other laboratory measurements that have been shown to predict anastomotic leak were also obtained. Inflammatory marker (e.g., white blood cell count, C-reactive protein) levels were obtained for patients when risk of leakage was even slightly suspected as there has been evidence that elevation of the C-reactive protein (CRP) level can be predictive of anastomotic leak complications (27–29). When multiple measurements were taken for a same patient, the highest value was used. In addition, Noble et al. showed that a combination of multiple laboratory values [the NUn score = 11.3894 + (0.005 × CRP) + (WCC × 0.186) – (0.174 × albumin); WCC refers to white blood cell count] can serve as a strong predictor of anastomotic leaks and other complications in esophageal resection (30); the NUn score was also obtained.

### Statistical Analyses

Data were analyzed using IBM SPSS Statistics 24.0 (SPSS, Inc., Chicago, IL, USA). Continuous data were represented by their mean and standard deviation and the categorical data as frequencies and percentages. Student's *t*-test was used to compare continuous variables, and the χ^2^ test and two-tailed Fisher's exact test were used to compare categorical variables. The average CRP values of postoperative days one, four, and seven were calculated for each treatment arm and plotted in a line graph.

## Results

**Figure 2:**
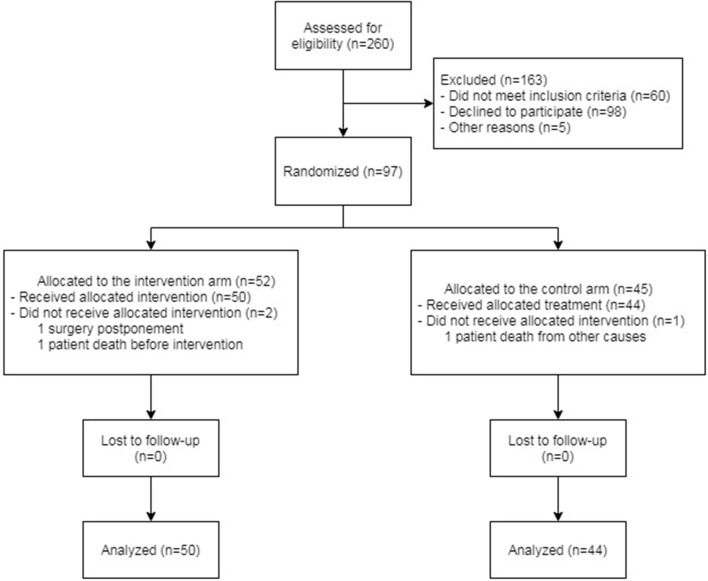
Flow diagram of patient enrolment.

A total of 94 patients were analyzed in the study. Of these patients, 50 were included in the intervention arm and 44 in the control arm. The scheme of enrollment is shown in Figure 2.

Patient demographics including age, sex, body mass index (BMI), comorbidities, American Society of Anesthesiologists (ASA) score, Eastern Cooperative Oncology Group (ECOG) performance status, type of reconstruction received, and the extent of lymphadenopathy are described in Table 1. There were no significant differences in these baseline characteristics between the two groups.

**Table 1:** Baseline characteristics of patients.

**Characteristics**	**Control**	**Biomaterial reinforcement**	***P***
	**(*n* = 44)**	**(*n* = 50)**	
Age (years)(SD)	61.7 (10.6)	59.9 (10.4)	0.402
Sex ratio (M:F)			0.87
Male	28 (63.6%)	31 (62.0%)	
Female	16 (36.4%)	19 (38.0%)	
Body mass index (kg/m^2^)	23.53 (2.93)	23.65 (2.92)	0.85
Comorbidities			0.225
None or 1	30	28	
>2	14	22	
Types of reconstruction			0.254
Billroth I	27	37	
Billroth II	6	7	
Esophagojejunostomy	11	6	
Extent of lymphadenopathy			0.694
D1+a	2	1	
D1+b	14	14	
D2	28	35	
T stage			0.365
T1a	12	18	
T1b	11	12	
T2	8	9	
T3	6	9	
T4a	7	2	
N stage			0.55
N0	22	32	
N1	11	8	
N2	5	6	
N3a	3	3	
N3b	3	1	
ECOG			0.544
0	41	48	
1	3	2	
ASA			0.231
1	2	5	
2	42	43	
3	0	2	

### Comparison of Clinicopathologic Outcomes

Postoperatively, data related to clinical outcomes including operation time, length of hospital stay, proximal resection margin, and complication rates were compared between the two groups (Table 2). There were no significant differences in the proximal resection margin, length of hospital stay, and operating time. The Clavien-Dindo (C-D) classification system was used for the comparison of complication rates. Of 44 patients in the control arm and 50 patients in the intervention arm, 19 (43.18%) and 20 (40.00%), respectively, experienced complications. No patients experienced complications of C-D class IIIb or higher. Although the control arm showed a tendency toward more severe (grade II or IIIa) complications, the difference was not statistically significant (*p* = 0.1157).

**Table 2:** Comparison of clinicopathologic outcomes.

	**Control no. (%)**	**Biomaterial reinforcement no. (%)**	***P***
Proximal resection margin (cm)	4.45	4.47	0.974
Length of stay (days)	15.48	13.38	0.142
Postoperative fever			0.385
≥1 Fever episode	16 (36.4%)	14 (28.0%)	
No fever	28 (63.6%)	36 (72.0%)	
Operation time (minutes)	185.91	164.90	0.060
C-D class			
0	25 (56.8%)	30 (60.0%)	
I	6 (13.6%)	12 (24.0%)	
II	9 (20.5%)	5 (10.0%)	
IIIa	4 (9.1%)	3 (6.0%)	
IIIb	0	0	
IVa	0	0	
IVb	0	0	
V	0	0	
Total	44	50	
0 + I	31 (70.5%)	42 (84.0%)	0.1157
II + III	13 (29.5%)	8 (16.0%)	

### Comparison of Anastomotic Site Leakage Complications

The results of the comparison of anastomotic leak rates are shown in Table 3. Two of 44 patients in the control group developed leakage during the 6-month follow-up period, whereas one of the 50 suffered leakage in the intervention arm during the same period. The patients in the control arm developed 13 and 6 days after surgery; the patient in the intervention arm developed leakage 18 days after surgery. This difference did not have a statistically significant value (*p* = 0.598). There were also no significant differences in the number of episodes of fever, the clinical sign most commonly used to suspect leakage after gastrointestinal surgery. Postoperative CRP levels, on the other hand, were found to be significantly lower in patients who received acellular dermal matrix reinforcement. Comparison of average CRP levels of each patient showed that the control arm had an average CRP level of 128.77 mg/dL, compared to the average of 77.38 mg/dL in the intervention arm. The NUn score, developed by Noble et al. (30) to predict anastomotic leak in esophageal resection patients using postoperative CRP levels, white blood cell counts, and albumin levels, also showed a statistically significant higher likelihood of progression to leakage for the control arm, with average scores of 13.29 (SD 0.667) and 12.71 (SD 0.667) for the control and intervention arms, respectively.

**Table 3:** Comparison of results related to anastomotic leakage.

**Variables**	**Control**	**Biomaterial reinforcement**	***P***
Leak found on imaging	2 (4.55%)	1 (2.00%)	(Fisher's) 0.598
Average CRP	128.77 (*n* = 17) (SD 97.08)	77.38 (*n* = 19)(SD 49.08)	0.049
NUn score	13.28 (*n* = 12) (SD 0.67)	12.71 (*n* = 13)(SD 0.67)	0.042

## Discussion

The primary endpoint of this study was clinically discovered episodes of anastomotic leakage. However, because we expected the number of these episodes to be low and our aim in this pilot investigation was to see the potential effect of acellular dermal matrix on postgastrectomy patients, clinical and laboratory measures that have been shown to predict anastomotic leaks were collected as secondary endpoints. These include cases of postoperative fever, inflammatory marker levels (i.e., CRP), and a scoring system developed to predict leaks in esophageal resection patients (i.e., NUn score). The severity of postoperative complications, as graded by the Clavien-Dindo classification system, was also collected as a secondary endpoint.

Acellular dermal matrix reinforcement of the anastomotic line after gastrectomy did not result in statistically significant improvements in either the occurrence of anastomotic site leakage. In addition, in terms of overall complication rates as represented by the Clavien-Dindo postoperative complication severity scale, there were no significant differences between the two treatment arms. However, the laboratory markers that reflect the likelihood of leakage, i.e., postoperative CRP levels and the NUn score, were significantly lower in the intervention arm.

The major limitation of our study is the small sample size. Because of the low rate of leakage complications, a large sample size—more than 1,500 patients in each arm based on the findings of this pilot investigation—is required for enough statistical power to demonstrate leak rate differences between the two arms. While an expected weakness, this pilot study is obviously underpowered to detect differences in clinical leak rates, and we have had to rely on extrapolation from secondary endpoints to draw conclusions about the effect of acellular dermal matrix reinforcement of the anastomotic site. However, the potential significance of our findings is discussed below, detailing findings that can serve as a reference for future studies of treatment for anastomotic leakage.

The most notable outcome of this investigation was the discrepancy in postoperative inflammation levels reflected by the CRP levels and NUn scores. Lower levels of these indicators have been shown to reflect lower likelihood of progressing to anastomotic leak (27–30). Studies have also shown that there is a correlation between overall postoperative complication rates and postoperative CRP levels as well (17, 31–33). Therefore, the reduced inflammatory levels in the acellular dermal matrix reinforcement group of this study may suggest that the anastomotic site reinforcement makes the operation more tolerable for the patient and has the potential to decrease likelihood of anastomotic leak development. In addition, the NUn score was specifically included in the analysis because while CRP is a nonspecific marker of inflammation and even CT images for leakage can be uncertain, the NUn score is a marker that is specifically designed to assess risk of leakage in the foregut. As such, it shows much less variance in either group compared to that of the nonspecific CRP. The lower NUn score in the intervention arm substantiates CRP findings. However because it is unknown how postoperative inflammation relates to each specific complication such as leakage, it must be noted that it is premature to firmly conclude on the clinical ramifications of lessened inflammation from these results alone.

Synthetic material reinforcements (as opposed to the biologic material acellular dermal matrix employed in this investigation) to anastomotic sites have been investigated in previous studies with positive results in reducing anastomotic complications. This material is already adopted for gastrectomy procedures. Similar to our investigation, Gayrel et al. have noted lower CRP levels in patients who received synthetic material reinforcement after sleeve gastrectomy than in those who did not, but this difference was without statistical significance (9). To the best of our knowledge, our investigation is the first to clinically show that a reinforcement material is able to statistically significantly reduce postoperative inflammation in upper gastrointestinal surgery. This may be related to the fundamental physiologic differences between biologic and synthetic materials. Acellular dermal matrix such as one used in this investigation is derived from human skin and treated with AlloClean® technology to leave only the extracellular matrix three-dimensional structure of the dermis. Synthetic buttress material, such as Gore Seamguard®, is made of polyglycolic acid:trimethylene carbonate (PGA:TMC) to form an interconnected pore structure to allow for cell infiltration and growth. Descriptions of both materials suggest they consist of comparable structures designed to perform similar functions. Synthetic buttress material is more integrated into the practice of current surgeons, but there were previous studies that have suggested superior performance of acellular dermal matrix over synthetic mesh reinforcement for hernia repairs and chest wall constructions (13, 14). A laboratory investigation found less inflammatory response in the integration of biologic materials compared to synthetic materials into tissue (34). We also speculate that the physiologic characteristic of biologic material has reduced postoperative inflammation as it leads to faster recovery of the anastomotic site, conferring a higher degree of protection against the development of anastomotic site weakness. However, we cannot yet draw conclusions on the difference between acellular dermal matrix reinforcement and synthetic reinforcements in the setting of gastrointestinal surgery, as no formal comparison between the two materials have been published to date.

In conclusion, this study was unable to detect any differences in leak rates or complication severity levels as measured by the Clavien-Dindo classification after application of acellular dermal matrix to reinforce the anastomotic site in patients who have undergone total or subtotal gastrectomy. The only notable difference between the intervention arm and the control arm was in the levels of postoperative inflammation. Findings from previous studies suggest that this decreased level of postoperative inflammation leads to more positive surgical outcomes in patients who received the biomaterial reinforcement, as these postoperative inflammatory marker levels are predictors of anastomotic leaks among other postoperative complications. Follow-up studies with larger sample sizes and longer follow-up periods may yield clinically significant differences in actual leak rates as well. In addition, further investigations into the mechanism behind postoperative inflammation after gastrointestinal surgery and the reason behind the decrease in inflammation with reinforcement of the anastomotic site with buttress materials may also uncover previously undiscovered surgical physiology.

## Data Availability Statement

The raw data supporting the conclusions of this manuscript will be made available by the authors, without undue reservation, to any qualified researcher upon request.

## Ethics Statement

The studies involving human participants were reviewed and approved by Korea University Institutional Review Board. The patients/participants provided their written informed consent to participate in this study.

## Author Contributions

SP first conceived, set up the parameters of the study, and was responsible for the oversight of the investigation as well as the manuscript production. He was also the surgeon responsible for operations detailed in the investigation. WK and CL further defined the patient enrollment criteria and data collection methods of the study, conducted the data analysis, and produced the final manuscript. LA and J-HK assisted in patient enrollment and led data collection. All authors contributed substantially to the study design and data analysis, revised the manuscript, and approved the current version of the paper. All authors contributed crucial intellectual information that made the production of this manuscript possible.

### Conflict of Interest

The authors declare that the research was conducted in the absence of any commercial or financial relationships that could be construed as a potential conflict of interest.
